# Psychological Underpinnings of Trust and Privacy Perception in the Medical Metaverse

**DOI:** 10.1089/jmxr.2023.0010

**Published:** 2024-09-26

**Authors:** Susan Persky

**Affiliations:** Social and Behavioral Research Branch, National Human Genome Research Institute, National Institutes of Health, Bethesda, Maryland, USA.

**Keywords:** medicine, privacy, psychology, immersive technology

## Abstract

The idea of a medical metaverse highlights the ways that immersive technologies will provide direct experiential access to the future of digital medicine, enabling clinical interactions, therapeutic treatment, and access to educational and support resources in persistent, multi-user, immersive digital environments. There are several points of tension, however, between the need for trust and privacy in the medical context and established norms and capabilities of the digital platforms on which health care is expected to increasingly take place. Considering trust and privacy experiences though a psychological lens, focused on tendencies, norms, and expectancies within digital environments can be instructive in considering these challenges. Here, the interface between human psychology and visions for the medical metaverse are discussed in the context of the environment, the self, interpersonal interactions, and group dynamics. Incorporating well-established psychological principles into user-centric design of future medical metaverse applications can enable us to take full advantage of emerging technological capabilities while building our digital interface with the future of medicine that is psychologically and ethically sound.

## Introduction 

The “metaverse” has risen and fallen in the hype cycle, but its core components continue to evolve and, whatever we ultimately call them, will transform the way we interface with health and medicine. Immersive technologies are central to this vision, promising direct experiential access to the digital future of medicine wherein persistent, multiuser, digital environments host clinical interactions, medical treatment provision, and access to health resources. For example, imagine your future avatar leaving the digital workplace to visit a physician in a virtual clinic space, followed by immersive, AI-guided physical therapy, followed later in the evening by an immersive virtual support group. In each of these settings, trust in providers and medical systems is critical for effective care, and preservation of patient privacy is both a key facet of trustworthiness and its own social good. While the “medical metaverse” (MM) does not yet exist, it is critical to consider potential points of tension between the need for trust and privacy on one hand, and established norms and capabilities of the digital platforms on which health care is expected to increasingly run. This can enable more thoughtful design of MM systems as they begin to emerge in earnest. This is especially important as elements of the MM have been projected to increase access to underserved and vulnerable populations where trust is already low and privacy violations may be of higher consequence. Through examination of longstanding human psychological and social tendencies and projection of how these tendencies will interface with emerging metaverse technologies with their own norms and practice, we can identify several points of tension to consider.

As humans transition from the physical world to 2D web and mobile technologies, to immersive, social, and 3D ones, we bring with us preexisting habits, heuristics, beliefs, norms, and expectations molded through evolutionarily-influenced predispositions and real-world experiences that may operate suboptimally in the MM. The *privacy paradox* (see Table 1 for definition of italicized terms) notes that while most technology users report highly valuing data privacy and fear the risks of unintended disclosure, they seldom take strong measures to protect their digital privacy.^1^ This constellation of ideas suggests that humans are very attuned to potential privacy invasion by other individuals as has been the primary privacy concern though human social history, however, humans tend to be less sensitive to privacy threats in modern digital ecosystems. Here, personal data are collected by faceless digital platforms and intermediates (e.g., tech companies), sometimes retained indefinitely. This paradox exemplifies the nature of conflicts between human psychology and the likely realities of trust and privacy in facets of the MM.

**Table 1.** Psychology Terminology Glossary

Term	Definition
The Privacy Paradox	A set of discrepancies in attitudes about the desire to have privacy online and ubiquitous engagement in behaviors that risk exposure or share personal data outright.
Attentional vigilance	Heightened alertness to detection of particular cues in the environment, often ones that signal various forms of potential threat.
Social scripts, schemas, and norms	Cognitive frameworks to help us organize information about the social world. Scripts and schemas refer to stories or mental models of how events typically unfold. Norms refer to what is deemed acceptable within a social group.
Cognitive priming	Exposure to a representation of an idea or feeling makes subsequent mental accessibility and application of that idea or feeling more likely.
Extended Self Theory	The notion that individuals define themselves in part by their possessions and elements of their online presence.
Embodiment	Connection between the mind and the physical or digital body which exert reciprocal influence on each other. In an immersive digital setting, related to a feeling of owning and controlling an avatar body.
Empathy	The capacity to understand and relate to the feelings of another person.
Positive emotion	Feelings that are pleasant or satisfying. Often linked to enhanced social connection and well-being.
Online disinhibition effect	Tendency to display more uninhibited behavior while in online (versus face-to-face) interaction.
Nonverbal behavior	Expression or communication with body language, facial expression, gestures, and other non-linguistic channels.
Nonverbal leakage	Unintentional, non-conscious expression of attitudes, beliefs, or emotions through nonverbal behavior. Often when there is a discrepancy between what someone is saying and what their nonverbal behavior is conveying.
Self-regulation	The ability to control and manage mental processes like thoughts, emotions, and behavior to achieve personal or social goals in a situation.
Social Identity Theory	The development of one’s self-identity rests in part on membership in social groups (e.g., religious groups) and categories (e.g., gender). From these memberships, individuals can derive self-esteem and other mental benefits.
Deindividuation	A state wherein individuals focus less on their own actions and feel less personally accountable due to a focus on group identity and loss of individual awareness.
Social Identity Model of Deindividuation Effects	A model that predicts when individuals are particularly likely to feel depersonalization within a group and how group norms and dynamics influence activities that result.
The Proteus Effect	Wherein individuals’ perceptions and behavior are shaped by the characteristics of their own avatars as well as the avatars of others.
Social influence	A constellation of processes through which individuals or groups influence the beliefs, attitudes, and behaviors of others (e.g., persuasion, conformity).
Presence	A subjective feeling of being physically and psychologically in a location, where that location may be a virtual environment made up of digital stimuli.

## Digital Environmental Factors

The nature of the physical environment has long provided individuals with cues as to when and whether our behavior and communication is private versus subject to surveillance. Psychological processes that help individuals understand these states range from automatic *attentional vigilance* to others’ gaze direction (cues to surveillance), and the development of *social scripts, schemas, and norms* representing expectation and agreement on the social nature of spaces (e.g., bathrooms as relatively private and drugstores as public). Digital places, such as those in the MM, will accordingly appear as sources of trust and privacy-relevant information, though they lack a link between appearance and reality. For example, immersive medical environments mimic real-world privacy cues such as simulating enclosed exam rooms where privacy, trust, and disclosure are the norm. Ironically, 3D spaces in the future MM will resemble the physical environments in which privacy intuitions evolved much more so than current 2D counterparts. Digital 3D spaces also readily integrate features like impressive diplomas that *cognitively prime* individuals to expect and perceive trustworthy behavior.^2^ Given the variety of entities that may function within the MM (e.g., hospital systems, health-focused corporations, tech companies), governed by a patchwork of policies and regulations, the true nature of trustworthiness and privacy preservation in any given space may be opaque.

## The Self

One widely touted benefit of immersive interactions, such as those in the MM, is the ability to create avatars to digitally represent us, whether a faithfully constructed digital twin, or a protective disguise to facilitate anonymity. Perceptions of personal anonymity are a powerful influence on behavior, including effects on the desire and likelihood to engage in non-normative behavior (sensitive disclosures, antisocial behavior). In a health context, for example, users of digital support groups report using avatars as a strategy to mask identity, which mentally “frees” them to share true thoughts and feelings with the group.^3^ In contrast to avatar disguises, *Extended Self Theory*^4^ suggests that constructed avatars and digital possessions can be an integral part of self-concept and identity expression. Customized, detailed digital content as self-expression can inadvertently reveal more than intended about real or idealized personal features. Other avatar features such as *embodied representation* (perception of being located “inside” of and directly controlling an avatar) can heighten the influence of environmental features that engender *empathy* or *positive emotion*,*^5^* making individuals more prone to perceive interactions as trustworthy or nonthreatening.^6^ As such, avatar representation can make individuals feel engaged and in control of their digital identity in several important ways, while in reality such control may be largely an illusion.

## Interpersonal Interactions

Activities in the MM will almost certainly involve a great deal of interpersonal interaction, and in these contexts too, the interface between features of the digital scenario and human psychology presents a mismatch. The *online disinhibition effect* has endured since the early days of computers, wherein individuals tend to be more open and honest about sensitive topics in computer-mediated interactions than real-world ones.^7^ Such disinhibition can be beneficial in contexts such as substance use counselling, but can also lead to unintentional or impulsive sharing in environments where assumptions about anonymity or privacy are not met. Other channels of disclosure in real-world settings are more subtle. Regardless of one’s words, true thoughts and feelings can nonconsciously emerge and be detected though *nonverbal behavior* and related channels like voice tone. In real-world environments, individuals can sometimes control or conceal this information “*leakage*” through effortful *self-regulation*. In the MM, however, the number of channels that convey personal information and the ability to extract meaning from them is greatly magnified through features such as eye tracking, face tracking, and sensors embedded in immersive displays. The longitudinal combination of these data streams is a unique consequence of immersive systems that provides unprecedented access to personal data. These data can readily be modeled to identify markers of emotional state, risk-taking tendencies, and to predict future behavior, even when individuals themselves are unaware of these states.^8^ At present, such capabilities are inconsistent with user perceptions; a survey of consumer attitudes toward biosensing technologies rated VR headsets as among the lowest risk for revealing personal information.^9^

## Group Interactions

In multiuser contexts, the psychology of groups can also increase the likelihood that MM users will place trust where it is unwarranted or engage in unintentionally self-disclosing communication and behavior. *Social Identity Theory*, for example, highlights how individuals are motivated to deepen ties to groups (e.g., health support groups) when they derive self-esteem and value from group participation. A key approach to strengthening social ties is through demonstration of interpersonal trust in the group setting (e.g., through self-disclosure) and adhering to group norms which may be set or influenced by underlying entities with varying motives. A twist on this phenomenon occurs when the group unit becomes more salient than the self, resulting in *deindividuation*—a temporary loss of self-identity as in the *Social Identity Model of Deindividuation Effects*.^10^ In real world and web-based settings, deindividuation encourages participation in behaviors that most individuals would be unlikely to enact, including antisocial activities (e.g., bullying, doxing) and impulsive behaviors that might put the self at risk. Environmental features in the MM could be readily leveraged to increase perceptions or likelihood of group cohesion making these outcomes more likely. For example, characteristics of focal avatars can be manipulated to increase their *social influence* capabilities (e.g., *the Proteus Effect*),^11^ potentially eliciting unearned trust.

## The Physical Environment

While participating in the MM, the digital becomes reality and one’s real-world physical environment becomes far less psychologically salient. This is described by the psychological concept of *presence*. Realistic, interactive digital environments such as those imagined for the MM will almost certainly be associated with high levels presence. In a practical sense, therefore, individuals immersed in MM environments will have low situational awareness and could be unknowingly observed in the physical environment while, for example, making disclosures to digital interaction partners. Bystanders in the physical environment may also experience privacy loss when microphones, cameras, and other sensors integrated into immersive hardware capture real-world information without providing clear cues to surveillance.

## Psychology Meets MM Design

As discussed elsewhere, education, policy, and legislation are crucial elements for a trustworthy and private MM but are unlikely to fully address the complexities of issues explored here. Approaches like implementing simple privacy settings and strong default protections should be considered a baseline. Beyond this, incorporating well-established psychological principles into user-centric MM design can elucidate user expectations and design features that conflict.

Given the vast flexibility of digital environments, we might imagine creation of a new visual lexicon that makes visible the role of underlying entities, surveillance, and the nature of other digital actors. For instance, many current digital platforms include markers of verified personal identities, which is likely to continue into the MM, and could be expanded to disambiguate the nature of other social actors in the MM. While sharing true identities of others in a therapeutic group may not be desirable, it may be useful to designate that the same, consistent person is represented by a familiar-looking avatar and username. Visual markers such as shifting representation features could likewise prompt the memory that in some multiuser MM contexts there is no such guarantee. Similarly, surveillance and collection of personal data could be represented by a digital video camera, rendered in the virtual scene to better align users’ mental models with the reality of a given context, with perhaps occasional representation of user data flowing from the individual to the camera and beyond. To ensure privacy where it matters most, visual and auditory barriers like a “cone” or bubble of silence could explicitly signal protected spaces, and also further psychologically reinforce the distinction between private and public areas in the MM. Over time, with continued salience of relevant cues, the schemas and norms that govern MM behavior and disclosure may adapt to ignore irrelevant privacy and trustworthiness cues such that individuals internalize MM realities. It is also conceivable that visibility of data collection and travel might prompt individuals to choose more secure environments, and companies to adopt policies to provide those environments.

Technical solutions such as AI guardians or assistants have been discussed for privacy prevention and could further support self-monitoring and controlling information “leakage” in nonverbal and other information channels. Technical solutions, guardians of another sort, could alert users when the real-world, physical environment is no longer private at times when situational attention is low.

In all, it will take a combination of approaches to support privacy and ensure trust is well placed in the MM, where our preexisting belief and behavior systems collide with unexpected, and sometimes hidden features of complex emerging technologies to create risks and challenges. Building with human psychology in mind will require collaboration between technologists, artists, psychologists, ethicists and others to design digital health platforms for the MM that take full advantage of emerging technological capabilities while becoming psychologically and ethically sound.

## Abbreviation Used

MM: medical metaverse
